# Death of Sir Michael Foster

**Published:** 1907-02-02

**Authors:** 


### DEATH OF SIR MICHAEL FOSTER.

As we go to press we are informed that Sir Michael Foster, K.C.B., late M.P. for London University, died on Tuesday in London, after a few days' illness, in his seventieth year. Born at Huntingdon on March 8, 1836, he was educated at University College, London, and in 1869 became Professor of Physiology in that medical school. Appointed Professor of Physiology in Cambridge University in 1883, he was one of the most distinguished scientists of his time, a President of the British Association, a Fellow of the Royal Society, and for twenty years its Hon. Secretary. His text-book of Physiology has been universally adopted in our great medical schools, and he has been deservedly esteemed as one of the glories of contemporary British science.

Sir Edward Sassoon is to preside at a banquet in aid of St. John's Hospital for Diseases of the Skin at the Savoy Hotel on Friday, May 10.

